# Investigating the role of ASCC1 in the causation of bone fragility

**DOI:** 10.3389/fendo.2023.1137573

**Published:** 2023-06-30

**Authors:** Barbara Voraberger, Johannes A. Mayr, Nadja Fratzl-Zelman, Stéphane Blouin, Suma Uday, Robert Kopajtich, Marijke Koedam, Helena Hödlmayr, Saskia B. Wortmann, Bernhard Csillag, Holger Prokisch, Bram C. J. van der Eerden, Ahmed El-Gazzar, Wolfgang Högler

**Affiliations:** ^1^ Department of Paediatrics and Adolescent Medicine, Johannes Kepler University Linz, Linz, Austria; ^2^ University Children’s Hospital Salzburg, Paracelsus Medical University Salzburg, Salzburg, Austria; ^3^ Ludwig Boltzmann Institute of Osteology at the Hanusch Hospital of OEGK and AUVA Trauma Center Meidling, 1^st^ Medical Department, Hanusch Hospital, Vienna, Austria; ^4^ Vienna Bone and Growth Center, Vienna, Austria; ^5^ Department of Endocrinology and Diabetes, Birmingham Women’s and Children’s NHS Foundation Trust, Institute of Metabolism and Systems Research, University of Birmingham Edgbaston, Birmingham, United Kingdom; ^6^ Institute of Neurogenomics, Helmholtz Zentrum München, Neuherberg, Germany; ^7^ Institute of Human Genetics, School of Medicine, Technical University of Munich, Munich, Germany; ^8^ Laboratory for Calcium and Bone Metabolism, Department of Internal Medicine, Erasmus MC, Erasmus University Medical Center, Rotterdam, Netherlands; ^9^ Amalia Children’s Hospital, Radboudumc, Nijmegen, Netherlands; ^10^ Department of Neonatology, Kepler University Hospital, Linz, Austria

**Keywords:** ASCC1, SMABF2, bone fragility, mesenchymal stromal cell, muscular atrophy, osteoblastogenesis, adipogenesis, osteoporosis

## Abstract

Bi-allelic variants in *ASCC1* cause the ultrarare bone fragility disorder “spinal muscular atrophy with congenital bone fractures-2” (SMABF2). However, the mechanism by which ASCC1 dysfunction leads to this musculoskeletal condition and the nature of the associated bone defect are poorly understood. By exome sequencing, we identified a novel homozygous deletion in *ASCC1* in a female infant. She was born with severe muscular hypotonia, inability to breathe and swallow, and virtual absence of spontaneous movements; showed progressive brain atrophy, gracile long bones, very slender ribs, and a femur fracture; and died from respiratory failure aged 3 months. A transiliac bone sample taken postmortem revealed a distinct microstructural bone phenotype with low trabecular bone volume, low bone remodeling, disordered collagen organization, and an abnormally high bone marrow adiposity. Proteomics, RNA sequencing, and qPCR in patient-derived skin fibroblasts confirmed that ASCC1 was hardly expressed on protein and RNA levels compared with healthy controls. Furthermore, we demonstrate that mutated *ASCC1* is associated with a downregulation of *RUNX2*, the master regulator of osteoblastogenesis, and *SERPINF1*, which is involved in osteoblast and adipocyte differentiation. It also exerts an inhibitory effect on TGF-β/SMAD signaling, which is important for bone development. Additionally, knockdown of ASCC1 in human mesenchymal stromal cells (hMSCs) suppressed their differentiation capacity into osteoblasts while increasing their differentiation into adipocytes. This resulted in reduced mineralization and elevated formation of lipid droplets. These findings shed light onto the pathophysiologic mechanisms underlying SMABF2 and assign a new biological role to ASCC1 acting as an important pro-osteoblastogenic and anti-adipogenic regulator.

## 1 Introduction

The activating signal co-integrator 1 complex subunit 1 (*ASCC1*; MIM: 614215) gene encodes one subunit of the tetrameric activating signal co-integrator 1 (ASC-1) ribonucleoprotein complex including also ASCC2 (MIM: 614216), ASCC3 (MIM: 614217), and TRIP4 (MIM: 604501) (1). In general, signal co-integrators can act bidirectionally as either transcriptional coactivator or corepressor. Thereby, they are building multiprotein complexes with transcription factors and are able to stimulate or inhibit gene expression depending on environmental cues (2, 3) or in a tissue-specific way (4). Bi-allelic variants in *ASCC1* leading to a loss of function (LOF) have been reported in infants with autosomal recessive spinal muscular atrophy with congenital bone fractures 2 (SMABF2; MIM: 616867). This rare genetic motor neuron disease is characterized by decreased or absent fetal movements, severe neonatal hypotonia, respiratory distress, multiple congenital long bone fractures, and joint contractures and usually has a rapid lethal outcome. Congenital fractures are very rare and do occur due to osteogenesis imperfecta (OI), other heritable bone fragility conditions, or maternal trauma (5, 6). To date, 18 SMABF2 patients (17 with congenital bone fractures) from 13 unrelated families have been reported. This high frequency of fractures suggests an important role of ASCC1 in bone development and fragility. However, so far, the nature of the bone fragility and the underlying bone pathophysiology of this ultrarare musculoskeletal disorder are poorly understood.

The ASC-1 complex acts as a transcriptional coactivator by binding to the transcription factors serum response factor (SRF), activating protein 1 (AP-1), and nuclear factor kappa-B (NF-kB). In fact, both ASCC1 and ASCC2 are essential for AP-1 transactivation (1). Interestingly, several AP-1 transcription factors were recently reported to act as positive regulators of bone and matrix formation and osteoblast differentiation (7–9) and they are able to interact with RUNX2, the master regulator of osteogenesis (10). A comparison of gene expression profiles between *ASCC1*-deficient cells derived from SMABF2 patients and wild-type cells revealed that mutant *ASCC1* downregulates genes involved in neurodevelopment and bone development such as *TNFRSF11B*, *SERPINF1*, and *RASSF2* (6).

Interestingly, a genome-wide association study recently reported an association between genetic variants in *ASCC1* and osteoporosis and obesity in postmenopausal women (11). Osteoporosis and obesity have some features in common including the bone marrow mesenchymal stromal cells (MSCs) which are able to give rise to osteoblasts and adipocytes (12). Osteogenic differentiation of MSCs is tightly regulated by the expression of specific molecular markers, e.g., RUNX2, osterix, β-catenin, alkaline phosphatase (ALP), osteocalcin, and type I collagen (13, 14), and by important signaling pathways such as transforming growth factor-beta (TGF-β), bone morphogenic protein (BMP), and Wnt signaling, which converge at transcription factors, e.g., RUNX2 (15, 16). The process of adipogenic differentiation is typically characterized by the expression of key transcription factors PPARγ and CEBPA and at later stages that of fatty acid synthase (FASN), glycerophosphate dehydrogenase, and acetyl CoA carboxylase as well as formation of lipids (13). Of particular interest to the exploration of ASCC1-induced bone fragility is that osteoblastogenesis and adipogenesis share an inverse relationship during life; while at a young age, MSCs predominantly develop into osteoblasts, and at an older age, they differentiate more into adipocytes (17–19). This change in the balance between osteogenic and adipogenic differentiation has been linked to diseases such as osteoporosis and obesity.

Here, we report for the first time the microstructural analysis of a transiliac bone sample of a SMABF2 patient bearing a novel homozygous *ASCC1* deletion. We also conducted functional tests *ex vivo* to assess the effect of the mutation on a major bone formation pathway and on the osteogenic and adipogenic differentiation of *ASCC1* knockdown human mesenchymal stromal cells (hMSCs).

## 2 Materials and methods

### 2.1 Sample collection

Blood samples of the patient and both parents as well as a transiliac bone and skin sample of the patient were obtained postmortem using standard techniques. A transiliac bone sample of an age-matched infant was identified from an ethically approved postmortem study on sudden infant death and used as a control for comparison of histomorphometric and BMDD outcomes. A skin biopsy was taken from an age- and gender-matched healthy infant. Written informed consent was obtained from the patient’s and control’s parents/guardian.

### 2.2 Bone histomorphometry and quantitative backscattered electron imaging

The transiliac bone sample was analyzed by histomorphometry and quantitative backscattered electron imaging (qBEI) at the Ludwig Boltzmann Institute of Osteology (LBIO) in Vienna using standard procedures (20–22).

In short, the sample was fixed in 70% ethanol, dehydrated in graded series of alcohol, and embedded in methyl-methacrylate. Static histomorphometric parameters of bone formation and resorption, as well as adipocyte surface in the total bone marrow space, were evaluated in undecalcified 3–5 μm thick sections stained with Goldner’s trichrome using a light microscope (Axiophot, Zeiss, Oberkochen, Germany) equipped with a digital camera (AxioCam HRc, Zeiss, Oberkochen, Germany) (22). For assessment of bone marrow adipocytes, optical images (1.5 mm²) from the sections were taken (n = 3) and analyzed with an in-house custom-made macro for ImageJ (version1.53) using the BioVoxxel Toolbox (23) to detect semiautomatically the adipocyte and to calculate the adipose tissue area as a percentage of the total marrow area. The minimal size of an adipocyte was defined at 525 µm². Structural histomorphometric parameters were calculated from the qBEI image. The residual bone sample block was prepared for qBEI. The surface plane containing bone tissue obtained after grinding and polishing was coated by carbon prior to analysis with a field emission scanning electron microscope (FESEM Supra 40, Zeiss, Oberkochen, Germany) equipped with a four-quadrant semiconductor backscatter electron detector. The Zeiss Supra 40 was operated with an electron energy of 20 keV. The gray levels reflecting the mineral/calcium content were calibrated by the material contrast of pure carbon and aluminum. Gray-level histograms were deduced and transformed into calcium weight percent (weight% calcium) histograms. The entire cross-sectional area of the transiliac bone sample was imaged with a spatial resolution of 1.8 μm per pixel. From the acquired image, parameters for histomorphometric indices of mineralized bone structure and the bone mineralization density distribution (BMDD) were evaluated. The BMDD parameters were measured in trabecular and cortical compartments separately. For cortical bone, the arithmetic mean was calculated from both cortices. All obtained parameters were compared with the bone sample of the age-matched control, as well as to available pediatric reference values (21, 22).

### 2.3 Fibroblast cell culture and treatment

For the isolation and culture of skin primary fibroblast cells from the patient and the age- and gender-matched healthy individual, the skin samples were washed with 70% EtOH and 1× PBS. After that, they were cut into small pieces and placed in a culture dish containing Dulbecco’s modified Eagle’s medium (DMEM), supplemented with 10% fetal calf serum (FCS), 1% anti–anti (antibiotic–antimycotic; 10,000 U/ml), and 1% L-glutamine (200 mM). Fibroblasts grew out of the tissue within several weeks, the culture medium was changed once a week, and cells were split at 80% confluency. Before the treatment with specific recombinant proteins, passage-matched patient and control cells were serum starved. To induce the TGF-β/SMAD pathway, cells were treated with 10 ng/ml recombinant human TGF-β1 protein (Abcam, Amsterdam, NL) for 48 h. All cells were incubated at 37°C in a humidified incubator with 5% CO_2_.

### 2.4 Exome sequencing

Genomic DNA extraction from peripheral blood leukocytes was performed using magnetic beads with the chemagic™ Prime DNA Blood 7k kit on a chemagic™ Prime 4/8 instrument (both Perkin Elmer, Waltham, MA, USA) according to the manufacturer’s instructions. The DNA concentration was assessed using the Qubit™ dsDNA BR Assay Kit and fluorometer (both Thermo Fisher Scientific, Waltham, MA, USA). Exonic regions were enriched with the SureSelect Human All Exon 60MbV6 Kit (Agilent, Santa Clara, CA, USA) and sequenced as 100-bp paired-end runs on an Illumina HiSeq4000 platform (Illumina, San Diego, CA, USA). Read alignment was performed to the reference genome (UCSC build hg19) using Burrows–Wheeler Aligner v.0.7.5a (24). SAMtools v.0.1.19 (25) and GATK v.3.8 (26) were used for the detection of small insertions and deletions (<200 nt) and single-nucleotide variants. Copy number variants (CNVs) were called using The R package ExomeDepth v.1.1.10 (27). For variant filtering and gene prioritization, a custom-build pipeline was used (28, 29).

### 2.5 RNA sequencing

Total RNA was isolated from patient-derived cultured skin fibroblasts with the RNeasy Mini Kit (QIAGEN, Hilden, Germany) according to the manufacturer’s instructions. The RNA integrity number (RIN) was assessed using the RNA 6000 Nano Kit and Agilent 2100 Bioanalyzer (both Agilent Technologies, Santa Clara, CA, USA). 1 µg of total RNA, quantified with the Qubit RNA BR Assay Kit and fluorimeter (both Thermo Fisher Scientific, Waltham, MA, USA), was used for library preparation with the TruSeq Stranded mRNA Sample Prep LS Protocol (Illumina, San Diego, CA, USA) described in detail in (30). RNA libraries were sequenced as 100-bp paired end runs on an Illumina HiSeq 4000 platform (Illumina, San Diego, CA, USA). After demultiplexing, read mapping to the hg19 genome assembly was performed with STAR v.2.7.0a (31) using default parameters. Detection of novel splice junctions was allowed by setting twopassMode to “Basic.” RNA reads per gene were counted using GenomicAlignments (32), and genes with a 95th percentile FPKM below 1 were considered as not expressed and removed from the dataset. Events of aberrant RNA expression were detected using the R package OUTRIDER (33) in a dataset of 269 samples.

### 2.6 Quantitative proteomics

Quantitative proteomics from cultured fibroblasts was performed as previously described in detail (34) with two minor changes. Peptides were labeled using Tandem Mass Tag (TMT) 11-plex, not 10-plex reagents (Thermo Fisher Scientific, Waltham, MA, USA). Peptide separation prior to mass spectrometric analysis was performed using high-pH reverse-phase and not-trimodal mixed-mode chromatography. Each TMT batch consisted of nine samples and two reference samples, which were kept identical between batches to allow for subsequent inter-batch data normalization. Protein expression outliers were computed by running one sample against all others in a moderated t test approach using the R/Bioconductor package limma (35) in a dataset of 71 samples from 8 individual TMT batches.

### 2.7 PCR and Sanger sequencing

A 130-bp PCR product was amplified using the following primers, which are flanking the deletion ASCC1-del-D (5′-gccaaactctttttcacagagg-3′) and ASCC1-del-R2 (5′-gctcggcttgttctgttttc-3′) to confirm the mutation in the patient-derived cells. In order to confirm an intact ASCC1 sequence in the control cells, a 215-bp PCR product was amplified using the following primers: ASCC1-del-D (5′-gccaaactctttttcacagagg-3′) and ASCC1-WT-R (5′-tggctaacaagcagaactgg-3′). The PCR was performed with Q5 Hot Start High-Fidelity 2x Master Mix (New England Biolabs, Frankfurt, Germany) and under the following thermocycling conditions: 30 s at 98°C followed by 31 cycles of 98°C for 10 s, 60°C for 30 s, and 72°C for 15 s, and another 7 min at 72°C. The amplification products were analyzed for their purity and size by agarose gel electrophoresis. After that, they were treated with Exo-CIP Rapid PCR Cleanup (New England Biolabs, Frankfurt, Germany) and sent to Eurofins for Sanger sequencing.

### 2.8 Quantitative PCR

Total mRNA from the cultured age-, gender-, and passage-matched patient and healthy control skin primary fibroblasts was isolated using Monarch^®^ Total RNA Miniprep Kit (New England Biolabs, Frankfurt, Germany). Reverse transcription (RT) was done by using High-Capacity cDNA Reverse Transcription Kit (Thermo Fisher Scientific, Vienna, Austria). Analysis of *ASCC1*, *RUNX2*, *SERPINF1*, *MMAA*, *CACFD1*, *RABEPK*, and *HPRT1* gene expression levels was performed on the LightCycler^®^ 480 System (Roche Diagnostics, Vienna, Austria) using LightCycler^®^ FastStart DNA Master HybProbe (Roche Diagnostics, #12239272001) and the following TaqMan^®^ probes: Hs00418608_m1 (ASCC1), Hs01051148_g1 (RUNX2), Hs00369340_m1 (SERPINF1), Hs02800695_m1 (HPRT1). Cycling conditions for quantitative PCR (qPCR) were 1 min at 95°C followed by 50 cycles of 95°C for 10 s and 60°C for 45 s. The delta–delta Ct method (2^–ΔΔCt^) was used to calculate the relative fold gene expression, whereby HPRT1 was used as a housekeeping gene.

Total mRNA isolation from hMSC and cDNA synthesis was performed as described in (36). In short, RNA extraction was done using TRIzol reagent and cDNA was synthesized using RevertAid First Strand cDNA Synthesis (Thermo Fisher Scientific). Real-time qPCR was performed on the QuantStudio™ 7 Flex Real-Time PCR System using Promega GoTaq™ qPCR Master Mix (Promega), and the primers are shown in **Supplementary Table 1**. Cycling conditions for qPCR were 10 min at 95°C followed by 40 cycles of 95°C for 15 s and 60°C for 1 min. The delta–delta Ct method (2^–ΔΔCt^) was used to calculate relative fold gene expression, whereby *36B4* was used as a housekeeping gene.

### 2.9 Protein isolation and Western blotting

Age-, gender-, and passage-matched fibroblasts from the patient and the healthy control, untreated and treated, were washed with cold 1× PBS and lysed by using RIPA buffer (Cell Signaling, Leiden, NL) supplemented with 100× protease inhibitor cocktail (Sigma-Aldrich, Vienna, Austria) and 100× phosphatase inhibitor cocktail (Sigma-Aldrich, Vienna, Austria) and 200× PMSF (Cell Signaling, Leiden, NL). Proteins were isolated by centrifugation at 14,000x g for 10 min at 4°C. Protein concentrations were measured using Pierce BCA Protein Assay Kit (Thermo Fisher Scientific, Vienna, Austria). Equal amounts of proteins (~30 µg) were loaded onto 4%–20% gradient gels (Bio-Rad, Vienna, Austria), separated by gel electrophoresis, and electrotransferred onto PVDF membranes (Bio-Rad, Vienna, Austria). After incubating the membranes with a blocking reagent (Bio-Rad, Vienna, Austria), they were probed with the following primary antibodies diluted in blocking buffer overnight: monoclonal anti-rabbit COL1A1 (1:1,000, Cell Signaling, Leiden, NL), polyclonal anti-rabbit COL1A2 (1:1,000, Abcam, Amsterdam, NL), monoclonal anti-rabbit actin (1:2,000, Cell Signaling, Leiden, NL), monoclonal anti-rabbit pSmad3 (Ser423/425, 1:1,000, Cell Signaling, Leiden, NL), and monoclonal anti-rabbit Smad3 (1:1,000; Cell Signaling, Leiden, NL). Membranes were washed with 1× TBS 0.1% Tween. Detection was carried out using the corresponding peroxidase-conjugated (HRP) secondary antibodies (1:15,000, Cell Signaling, Leiden, NL) diluted in a blocking buffer with incubation for 1 h at room temperature. The membranes were washed again with 1× TBS 0.1% Tween and developed using SignalFire Elite ECL Reagent (Cell Signaling, Leiden, NL). Quantification of the bands was done with Bio-Rad Image Lab 6.1 software to calculate the expression levels. All band intensities were normalized to those of actin.

### 2.10 Mesenchymal stromal cell differentiation cultures

Human bone marrow-derived mesenchymal stromal cells (hMSCs) were obtained from Lonza (#PF-2501, Donor #4266). HMSCs were cultured and differentiated as described by Bruedigam et al. (36). Briefly, cells were seeded (20,000 cells per well of a 12-well plate) and maintained for 2 days in alpha minimum essential medium (α-MEM, Gibco) supplemented with 10% heat-inactivated FCS, penicillin–streptomycin, HEPES, and calcium chloride and adjusted to a pH of 7.5. Osteogenic differentiation was initiated using a differentiation medium containing α-MEM supplemented with 10 mM β-glycerophosphate and 100 nM dexamethasone added for a period of 25 days. Adipogenic differentiation was initiated using α-MEM supplemented with 100 nM dexamethasone, 500 µM 3-isobutyl-1-methylxanthine, and 60 µM indomethacin for 17 days. Cells at passage 8 were used in all experiments, and the differentiation medium was refreshed twice a week. All experiments were performed in at least triplicates per condition.

### 2.11 Short hairpin-mediated knockdown of ASCC1 by lentiviral transduction

The two constructs of short hairpin RNAs (shRNAs) targeting endogenous ASCC1 ID12209 (renamed as #1 shRNA ASCC1) and ID12211 (renamed as #2 shRNA ASCC1) and the non-targeting shRNA with a scrambled sequence serving as a control (Scr) were purchased from the TRC-Hs1.0 library (Sigma-Aldrich) (**Supplementary Table 1** for sequences). Lentivirus (LV) was produced by transient transfection into 293FT cells using a standard calcium phosphate precipitation method as described previously (37). After 48 h, LV-containing supernatants were harvested and used immediately for hMSC transduction (24 h after attachment). One day later, the medium was replaced with osteogenic or adipogenic differentiation induction medium and cells were cultured until further analysis.

### 2.12 Calcium assessments

Calcium measurements and Alizarin Red S staining at day 25 of osteogenic differentiation were carried out as previously described (36). For the calcium quantification, cell extracts were prepared using PBS containing 0.1% Triton X-100 and further sonication. These samples and the residual extracellular matrix material in the plates were incubated overnight with 0.24 M HCl at 4°C. The calcium concentration was determined photometrically at 595 nm using a calcium assay reagent by combining reagent I (1 M ethanolamine buffer pH 10.6) with reagent II (0.35 mM O-cresolphthalein; 9.8 mM 8-hydroxyquinoline in 0.6 M HCl) in a ratio 1:1. Measurements were performed using the Perkin Elmer Victor X2 multilabel Microplate Reader. For the Alizarin Red S staining, cells were washed in PBS, fixed with ice-cold 70% ethanol for 1 h, and then stained for 5–10 min with a solution of 0.6% Alizarin Red S (pH 4.1).

### 2.13 Oil Red O staining

Oil Red O staining at day 17 of adipogenic differentiation was performed, as previously described (36). Briefly, cells were flushed with PBS, fixed with 10% formalin overnight at 4°C, and rinsed with 60% 2-propanol. The cells were then stained with Oil Red O working solution (three parts of saturated Oil Red O solution and 2 parts of Milli-Q water) for 10–30 min. Cells were further incubated with DAPI (100 µg/ml in PBS) for 5 min in the dark. Imaging of all the wells was done. For the extraction of the lipid droplets, the plates were dried and incubated with IGEPAL working solution (4% IGEPAL in 100% 2-propanol) for 30 min. The quantification of the lipid droplets was done by photometric measurements at 490 nm.

### 2.14 Statistical analysis

Results were assessed using two-sided Student’s t-tests to compare two groups and expressed as means ± SEM. A value of *P* < 0.05 was considered significant. All statistical evaluations were undertaken using GraphPad Prism 5 software.

## 3 Results

### 3.1 Clinical characteristics

**Figure 1:**
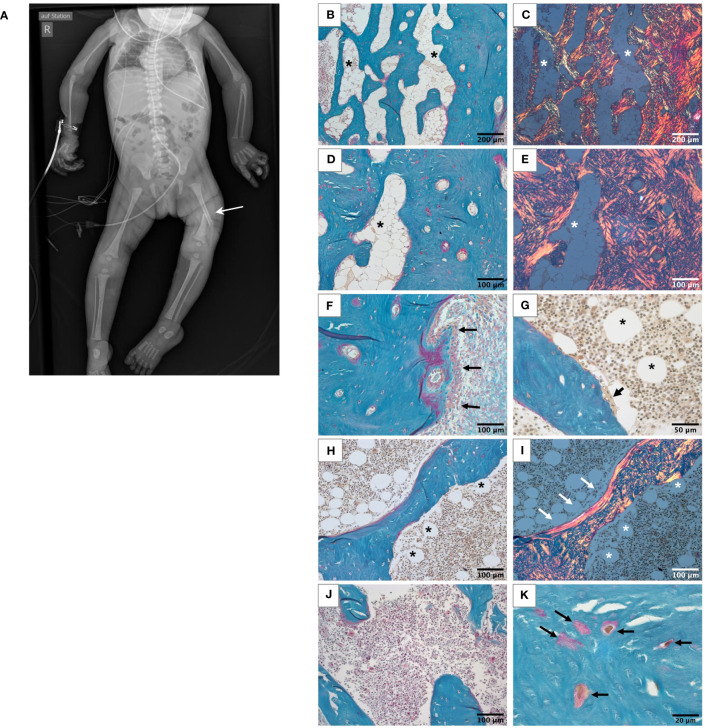
Skeletal phenotype of the affected patient. **(A)** Full-body X-ray of the patient showing gracile long bones, very slender ribs, and a left femur fracture (white arrow). **(B-I)** Histological features of the bone sample from the patient viewed in light microscope. All undecalcified sections, Goldner’s trichrome staining with the mineralized matrix in green, the unmineralized matrix, i.e., osteoid, in red. White roundish cells in the bone marrow are adipocytes (asterisks). **(B-E)** Highly trabecularized cortical bone. Identical regions viewed in transmission light **(B, D)** and in polarized light **(C, E)**, respectively. Note the highly disordered bone structure, characteristic for woven bone and the high number of adipocytes in the bone marrow compartment. **(F)** Outer periosteal surface covered by bone forming osteoblasts (black arrows). **(G)** Bone-resorbing multinucleated osteoclast on the trabecular feature (black arrow). **(H, I)** Trabecular feature viewed in transmission light **(H)** and polarized light **(I)**. Most of the bone tissue consists of woven bone as the cortex. Only a thin rim of lamellar bone covers one side of the trabecular feature (white arrows). Scattered adipocytes are visible in bone marrow of the trabecular compartment (asterisks), which are not present in the bone sample from the age-matched control **(J)**. Osteocyte lacunae in the mineralized bone tissue are irregularly shaped and distributed **(K)**.

This girl was born prematurely by cesarean section at 34 + 1 weeks of gestation to consanguineous parents of Pakistani descent. At birth, the patient demonstrated severe congenital muscular hypotonia, a virtual absence of spontaneous movements, inability to breath and swallow, and required cardiopulmonary resuscitation and ventilation. She acquired a left-sided femur fracture during the resuscitation process including an intraosseous needle placement at the tibia. Additionally, she had arachnodactyly, gracile long bones, and very slender ribs on X-rays (**Figure 1A**). Her parents were reported to be healthy except for hearing loss in the mother. At birth, the girl also had a severe vitamin D deficiency with hypocalcemia as a consequence of maternal vitamin D deficiency. Her calcium and vitamin D levels normalized promptly following supplementation. Additionally, she suffered transient thoracic chyle leak and intestinal hypomobility and developed progressive brain atrophy. Due to progressive respiratory insufficiency, the patient died at the age of 3 months, immediately after the withdrawal of care with parental consent.

### 3.2 Bone histology and histomorphometry

**Figure 2:**
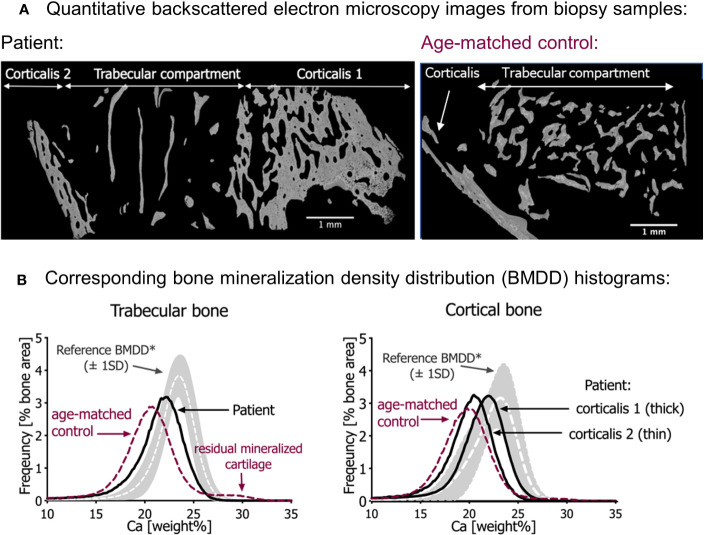
Quantitative backscattered electron imaging (qBEI). **(A)** qBEI overview of the transiliac bone samples of the patient (left) and the age-matched control (right). Note the scarcity of trabecular bone in the sample from the patient. **(B)** Corresponding bone mineralization density distribution (BMDD) in trabecular bone (left) and cortical bone (right). The gray bands represent the reference values (± SD) from Mähr et al. (21). Note that the BMDD curves from both infants are shifted to the left, toward a lower mineral content compared with the reference curves. In both bone compartments, the mineral content of the patient’s bone sample is however slightly increased compared with the age-matched control (red dotted line). Note in the trabecular bone of age-matched control sample an additional small peak at the right side of the BMDD curve representing highly mineralized residual cartilage. This peak is not visible in the patient’s bone sample.

Bone histology showed highly disordered collagen organization typical for primary woven bone and a remarkably high quantity of bone marrow adipocytes (**Figures 1B–I**), normally absent in infants since at birth bone marrow is entirely hematopoietic. However, bone marrow adipocytes were not homogenously distributed throughout the bone sample: the marrow area in the highly trabecularized cortical plates and toward the central trabecular area was nearly completely replete by adipocytes (**Figures 1B–E**), whereas scattered adipocytes were viewed in the central part of the trabecular compartment (**Figures 1G–I**). In total, the adipocytes covered 19.9% of the bone marrow area of the patient (**Table 1**). In contrast, no adipocyte was viewed in the sample of the age-matched control infant (**Figure 1J**). Active bone formation was observed on the periosteal surfaces (**Figure 1F**). Furthermore, irregularly shaped osteocyte lacunae were detected in the trabecular and cortical bone (**Figure 1K**) in accordance with the woven bone conformation (**Figure 1I**). Structural histomorphometric analysis revealed that trabecular bone volume was reduced by around 50% compared with reference values from older children, the age-matched control sample (**Table 1**), and previously reported infants (38). Osteoclasts were mostly viewed on cortical surfaces leading to the observed trabecularization of the cortical plate (**Figure 2A**), but since osteoblasts and osteoclasts were both highly decreased in the central trabecular area, there was no proper bone remodeling into lamellar bone (**Figures 2A**, **1G**). As a result, the thin and isolated trabecular features consisted mainly of immature woven bone rather than lamellar bone (**Figures 1H, I**). Despite the young age of the child, the bone sample did not show any residual mineralized cartilage.

**Table 1:** Bone histomorphometry results from the patient in comparison with reference data and control infant.

	Patient (age 3 months)	References values (22) (age range 1.5-6.9 years)	Control (age 3 months)
Structural histomorphometric parameters			
Bone volume/tissue volume [%]	9.6	17.7 ± 2.6	22.32
Trabecular thickness [µm]	50.5	101 ± 11	62.42
Trabecular number [1/mm]	1.89	1.77 ± 0.31	3.58
Cortical width [µm]	Mean: 1.10	0.70 ± 0.28	0.30 (only one cortex available)
Static histomorphometric parameters of bone formation and resorption			
Osteoid thickness [µm]	3.1	5.8 ± 1.4	3.31
Osteoid surface/bone surface [%]	25.3	34.0 ± 6.7	19.67
Osteoid volume/bone volume [%]	2.01	3.97 ± 1.19	2.64
Osteoblast surface/bone surface [%]	1.6	8.5 ± 4.1	Not evaluable
Eroded surface/bone surface [%]	6.2	14.8 ± 4.4	Not evaluable
Osteoclast surface/bone surface [%]	0.23	1.11 ± 0.75	2.02
Bone marrow adipocyte area			
Adipocyte area/marrow area [%]	19.9	Not defined	0

### 3.3 Bone mineralization density distribution

Compared with the reference values of healthy older children, the bone mineralization density distribution (BMDD) curves of the patient in both cancellous and cortical bone were shifted to the left, toward a lower mineral content of the matrix (**Figure 2B**, **Table 2**). This was in line with a reduced average degree of mineralization (CaMean) in both bone compartments. The fraction of lowly mineralized bone tissue (CaLow) was elevated in the cortical and trabecular bone, whereas the fraction of highly mineralized bone tissue (CaHigh) was below the normal range. However, bone matrix mineralization was slightly increased compared with the age-matched control bone sample consistent with the presence of woven bone observed by polarized light microscopy, which is known to have higher mineralization than lamellar bone. Thus, the combination of high woven bone and the increased matrix mineralization in the patient’s bone compared with the age-matched control suggests a failure of proper remodeling into mature lamellar bone and proper endochondral ossification.

**Table 2:** Results of qBEI analyses from the affected patient in comparison with reference values and control infant.

	Cancellous bone			Cortical bone			
BMDD parameters	Patient (age 3 months)	Reference values (21) healthy children (n=50)	Control (age 3 months)	Patient Corticalis1	Patient Corticalis 2	Reference values (21) children (n=50)	Control (age 3 months)
CaMean [wt% Ca]	20.34	22.48 (0.73)	19.88	20.31	19.09	21.86 (1.15)	19.02
CaPeak [wt% Ca]	22.01	23.39 (0.70)	20.62	21.83	20.45	22.67 (1.21)	19.76
CaWidth [Δ wt% Ca]	4.51	3.64 [3.47; 3.99]	4.85	4.68	4.51	4.07 [3.73; 4.68]	5.37
CaLow [% bone area]	16.22	5.57 [4.78; 6.80]	21.58	15.24	24.90	6.86 [5.06;11.48]	31.50
CaHigh [% bone area]	0.36	1.52 [0.62; 2.22]	4.22	0.37	0.02	1.01 [0.44;1.89]	1.65

Values are given as mean ( ± SD) or median with interquartile range [25%, 75%].

**Definition of BMDD parameters** (21):

CaMean: the average calcium concentration (weighted %Ca), CaPeak: the most frequently occurring calcium concentration (the peak position of the BMDD) in the sample, CaWidth: the width of the BMDD distribution (full width at half-maximum) reflecting the heterogeneity in matrix mineralization, CaLow: the percentage of low mineralized bone area, defined as mineralized below 17.68 wt% calcium, which corresponds to the fifth percentile of the reference BMDD in adult trabecular bone, CaHigh: the percentage of highly mineralized bone matrix, having the calcium content above 25.30 wt% calcium, which corresponds to the 95th percentile of the reference BMDD in adult trabecular bone.

Given this striking bone phenotype, of high bone marrow adiposity and low osteoblast number, and since osteoblasts and adipocytes are both derived from hMSCs but are reciprocally regulated, we hypothesized that the patient has an intrinsic defect at early stages of osteogenic differentiation.

### 3.4 Identification of a novel homozygous deletion in *ASCC1*

By exome sequencing, we identified a novel homozygous deletion in the *ASCC1* gene (MIM: 614215; *Homo sapiens* chromosome 10, GRCh38.p14 Primary Assembly NC_000010.11: g. 72138925_72163147del24223; NM_001198799.2: c. 574-1471_831-5742del24223). This deletion of around 24 kb within the 10q22.1 chromosome band encompasses exons 7 and 8 of the *ASCC1* gene (see integrated genome viewer (IGV) screenshot in **Supplementary Figure 1B**). The variant was absent from GnomAD (gnomad.broadinstitute.org) in homozygous and heterozygous states. Both parents were heterozygous carriers for the variant confirming recessive inheritance (**Supplementary Figure 1A, B**). The variant was additionally confirmed by Sanger sequencing in the patient (**Supplementary Figure 1C**). The variant is predicted to lead to a nonsense-medicated mRNA decay and subsequent absence of the functional protein.

### 3.5 Multi-omics analyses using patient fibroblasts showed profound downregulation of *ASCC1, RUNX2*, and *SERPINF1* expression

**Figure 3:**
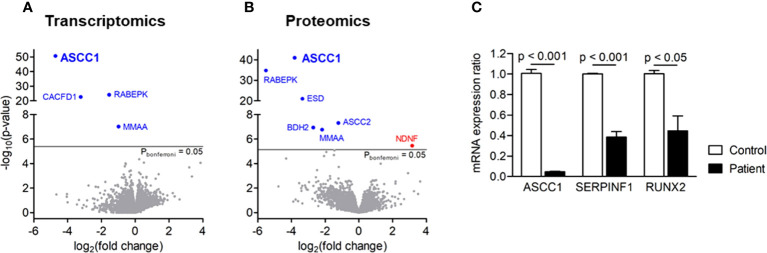
Gene expression in patient-derived skin fibroblasts. **(A)** Transcriptomics and **(B)** proteomics analyses showing significance (-log10(P), y-axis) versus fold change (log2(fold change), x-axis). Blue genes are significantly downregulated, and red genes are upregulated. **(C)** Quantitative PCR showing the mRNA expression ratio of *ASCC1*, *SERPINF1*, and *RUNX2* in age-, gender-, and passage-matched patient and control fibroblasts. Target genes were normalized to *HPRT1*. Columns represent the mean of at least three independent experiments, and error bars show the standard error of the mean (SEM). The *p* values were calculated using a two-tailed *t* test.

We analyzed the whole transcriptome and proteome in patient-derived fibroblasts by RNA sequencing and mass spectrometry and found *ASCC1* to be hardly expressed, either on RNA or on protein level (**Figures 3A, B**). Based on proteomics analysis, it was also shown that *ASCC2* is highly downregulated. Moreover, RNAseq and proteomics displayed significant downregulation of a few other genes apart from *ASCC1* such as *RABEPK* and *MMAA*. The downregulation of *MMAA, CACFD1*, and *RABEPK* at the RNA level was confirmed by qPCR (**Supplementary Figure 2**). Since these genes are not known to be associated with any bone disease or with *ASCC1*, they were not further analyzed. By qPCR, we confirmed the profound downregulation of *ASCC1* mRNA and showed that the expression of *RUNX2* and *SERPINF1* was reduced by more than 50% (p < 0.05; **Figure 3C**).

### 3.6 Patient fibroblasts exhibit a dysregulation of the TGF-β/SMAD signaling pathway

**Figure 4:**
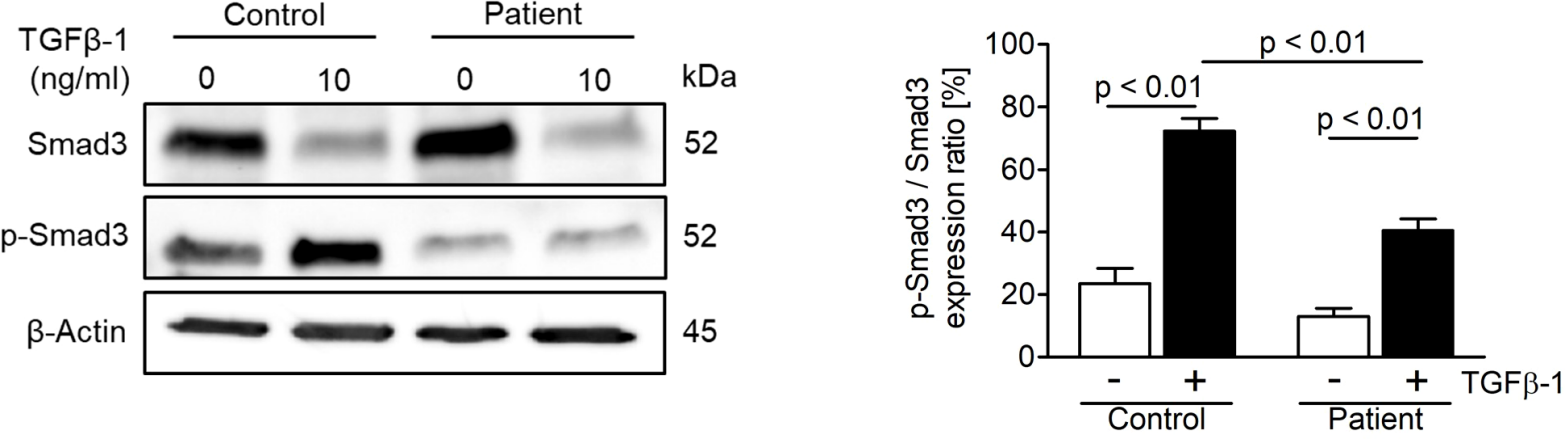
Analysis of TGF-β/SMAD signaling in age-, gender-, and passage-matched patient and control fibroblasts. Treating the cells with recombinant human TGF-β1 protein (10 ng/ml) for 48 h to activate the pathway showed a downregulation of phosphorylated SMAD3 over total SMAD3 in patient cells. The target proteins were normalized to β-actin. Columns represent the mean of three independent experiments, and error bars show the standard error of the mean (SEM). The *p* values were calculated using a two-tailed *t* test.

In order to study the effect of the novel *ASCC1* mutation on TGF-β/SMAD signaling, an important bone pathway and patient-derived and control fibroblasts were treated with the TGF-β1 ligand. This treatment lead to a dysregulation of TGF-β/SMAD signaling (**Figure 4**). The patient cells showed a significant reduction in the phosphorylation of SMAD3 of more than 40% (p < 0.01) compared with the treated healthy control cells. In addition, we analyzed the protein expression of type I collagen, which revealed no notable difference in COL1A1 or COL1A2 (**Supplementary Figure 2**).

### 3.7 Knockdown of *ASCC1* in hMSCs leads to reduced osteoblastogenesis and enhanced adipogenesis

**Figure 5:**
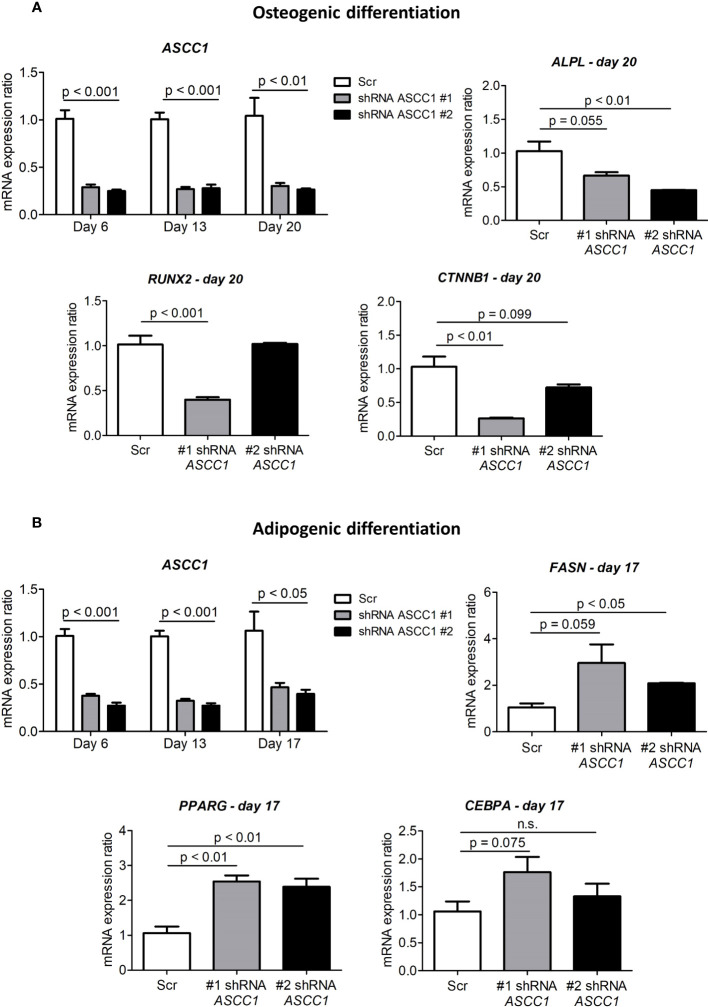
Gene expression analyses in the osteogenic and adipogenic differentiation of *ASCC1* knockdown hMSCs. **(A)** *ASCC1* and the osteoblast specific markers *ALPL*, *RUNX2*, and *CTNNB1* are substantially downregulated in *ASCC1* KD hMSC-derived osteoblasts. **(B)** *ASCC1* is downregulated and the adipocyte-specific markers *FASN*, *PPARG*, and *CEBPA* are upregulated in *ASCC1* KD hMSC-derived adipocytes. The relative mRNA expression ratio was calculated with the delta–delta Ct method using 36B4 as a housekeeping gene. Columns represent the mean of four biological replicates and show standard error of the mean (SEM). The *p* values were calculated using a two-tailed *t* test.

**Figure 6:**
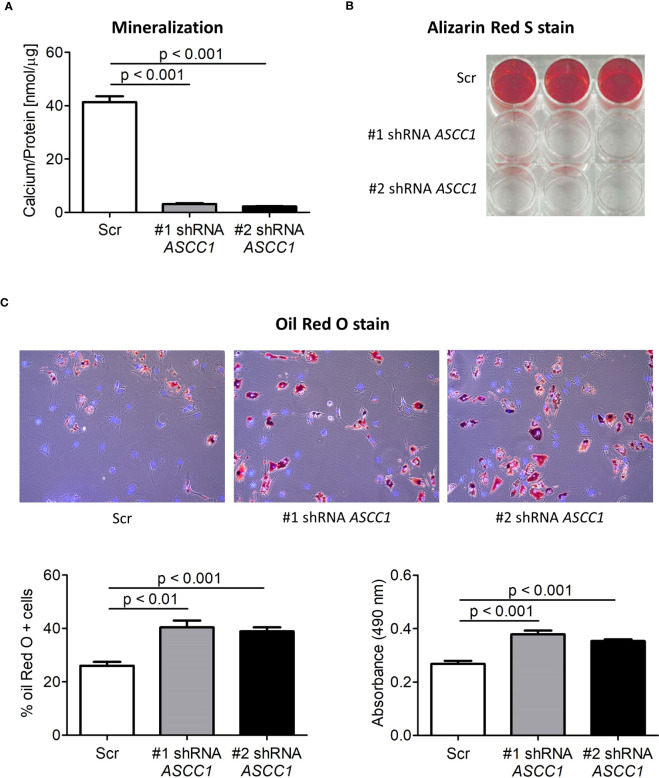
Biochemical analyses of the osteogenic and adipogenic differentiation of shRNA-mediated knockdown of *ASCC1* in hMSCs compared with scrambled control (Scr). KD of *ASCC1* induces a tremendous inhibition of **(A)** mineralization analyzed by quantifying the calcium content and **(B)** staining calcium deposits with Alizarin Red S on day 25 of differentiation. **(C)** KD of *ASCC1* enhances adipogenesis by stimulating the formation of lipid droplets as demonstrated by Oil Red O staining and quantification of lipid droplets on day 17 of differentiation. The three panels show lipid droplets in red and nuclei in blue. The two graphs present the percentage of Oil Red O positive cells and the measured absorbance at 490 nm of the extracted lipid droplets.

To determine the role of ASCC1 in human osteogenic and adipogenic differentiation, we used shRNAs targeting endogenous *ASCC1* to knock down its expression in hMSCs. Two separate shRNAs (#1 shRNA *ASCC1*, #2 shRNA *ASCC1*) substantially decreased *ASCC1* mRNA expression at day 6 of osteogenic and adipogenic differentiation by approximately 70% (p < 0.001) and 60% (p < 0.001), respectively (**Figures 5A, B**), compared with the scrambled control cells (Scr). *ASCC1* expression remained downregulated until the end of osteogenic and adipogenic differentiation (**Figures 5A, B**). While the knockdown of *ASCC1* resulted in the inhibition of osteogenesis, it promoted adipogenesis based on mRNA expression of osteogenic and adipogenic markers. *ALPL* and *RUNX2* using one of the two shRNAs and *CTNNB1* using shRNA #1 were substantially downregulated at day 20 of osteogenic differentiation (**Figure 5A**), whereas *FASN* and *PPARγ* revealed a pronounced upregulation in particular *PPARγ* with a 2.5-fold increase on day 17 of adipogenic differentiation (**Figure 5B**). The phenotype of restrained osteogenesis was reinforced by a massive reduction (>90%) in the amount of incorporated calcium in hMSC-derived osteoblasts at day 25 (**Figure 6A**). This was further confirmed by Alizarin Red S staining (**Figure 6B**). On the contrary, oil red O staining in hMSC-derived adipocytes at day 17 of differentiation showed an increased amount of Oil red O positive cells by approximately 50%. This enhanced adipogenesis was further underpinned by the photometric quantitative measurement of extracted lipid droplets, showing also an increase of around 50% (**Figure 6C**).

## 4 Discussion

**Figure 7:**
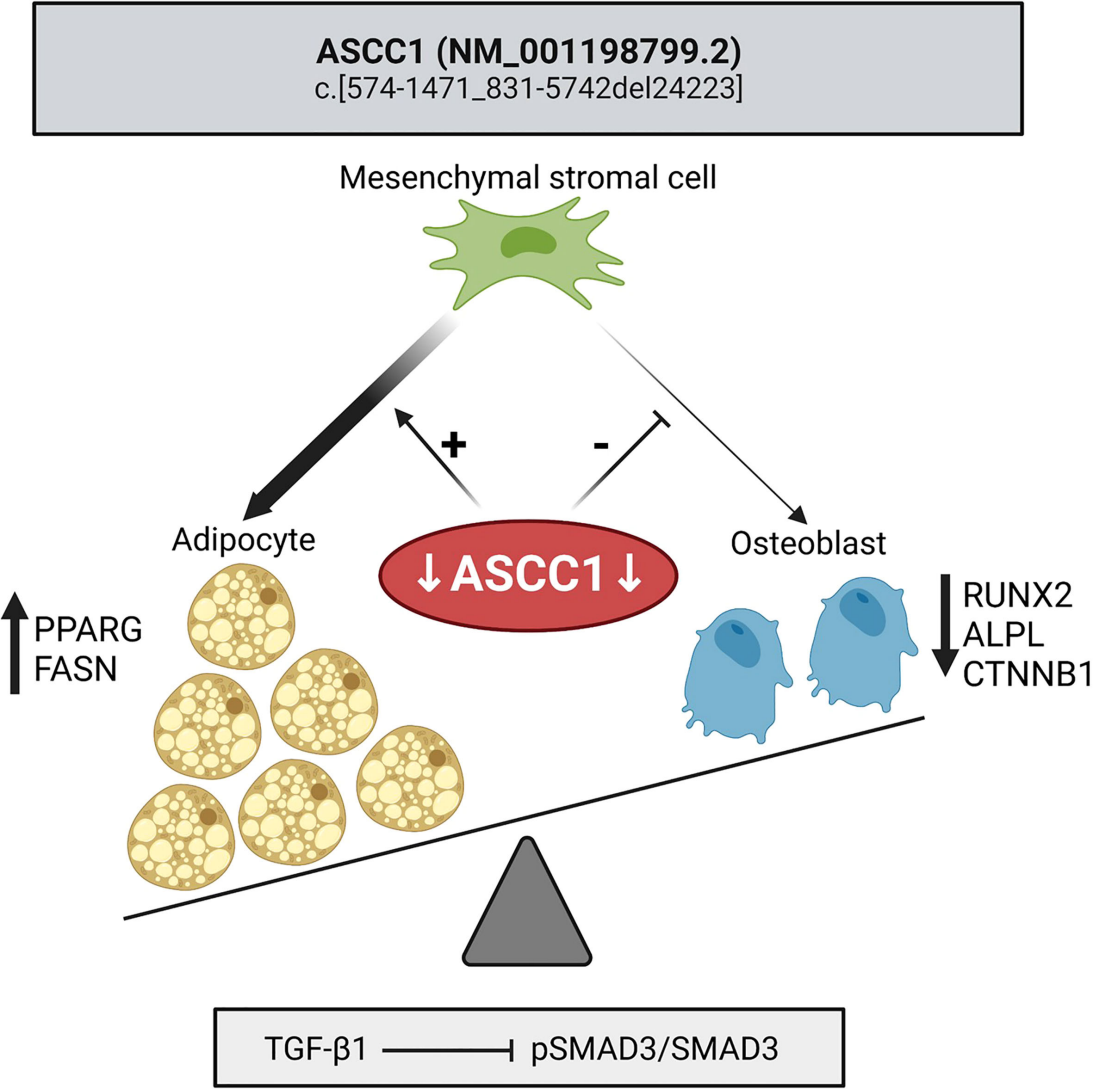
Mutant *ASCC1* directs MSC fate toward adipocytes by increasing adipogenic markers *PPARG* and *FASN* and away from osteoblasts by decreasing osteogenic markers *RUNX2*, *ALPL*, and *CTNNB1*. Mutant *ASCC1* inhibits TGF-β/SMAD signaling when stimulated with recombinant human TGF-β1, through downregulating the expression ratio of pSMAD3/SMAD3 in patient fibroblasts. Figure created with BioRender.com.

In this study, we demonstrate that knockdown of *ASCC1* in hMSCs resulted in inhibited osteoblast differentiation and stimulated adipogenesis (**Figure 7**) explaining the patient phenotypic characteristics with high bone marrow adiposity, decreased osteogenesis, and bone fragility. We thus uncover an important biological role of ASCC1 in the early stages of osteogenesis and adipogenesis. Moreover, the bone sample of the affected girl showed overall disordered collagen fibril organization (**Figure 1C, E, I**) due to the accumulation of immature woven bone, with irregularly shaped and positioned osteocyte lacunae, as typical for woven bone (**Figure 1K**) (39), low bone remodeling, low trabecular bone volume, and an abnormally high bone marrow adiposity. This striking bone fragility phenotype was underpinned by a dysregulation of TGF-β/SMAD and a downregulation of *RUNX2* and *SERPINF1*.

*ASCC1* encodes a protein of the transcriptional coregulator complex ASC-1, and recessive mutations cause the ultrarare bone fragility disorder SMABF2. All affected individuals reported to date exhibited similar phenotypes and prognoses, confirming a strong disease–gene relationship. However, to date little is known about the function and mechanism of ASCC1 or the entire ASC-1 complex in general and especially in the pathophysiological processes underlying SMABF2. Only one case was investigated on a cellular and pathomechanistic level, which revealed that mutant *ASCC1* downregulates genes associated with neurogenesis, neuronal migration/pathfinding, and bone development. The typical cause of death in SMABF2 is respiratory failure from severe muscle hypotonia. A zebrafish model demonstrated that knockdown of *ASCC1* disrupts α-motoneuron outgrowth and formation of myotomes as well as neuromuscular junctions (6). However, the disease obviously comes with a distinct bone fragility phenotype, the mechanism of which we set out to explore.

The deleted exons 7 and 8 in *ASCC1* in our patient encode part of the predicted protein kinase A (PKA) anchor protein nuclear localization signal domain of the ASCC1 protein, which is responsible for targeting the protein to the nucleus of the cell (40). Giuffrida et al. have previously reported a microdeletion of exons 7–10 and indicated that this mutation could lead to the complete absence of the protein within the nucleus where it acts as a transcription regulator (41). This is in agreement with our gene expression and proteomics analyses showing that ASCC1 is almost absent on RNA and protein levels. Additionally, these analyses indicated a significant downregulation/upregulation of other genes, which are not known to be associated with bone development or strength (details on gene function see **Supplementary Material**).

Our study is also the first to report the microstructural bone phenotype in an affected human. Trabecular bone volume and turnover were extremely low, and collagen fibrils appeared disorganized under polarized light microscopy as expected in the presence of woven bone, all contributing to a reduced mechanical competence of bone. Moreover, we observed very few osteoblasts and irregularly shaped and positioned osteocytes lacunae, but a high amount of bone marrow adipocytes which are usually absent in infants (17–19, 42). Since osteoblasts and adipocytes derive both from multipotent MSCs (12, 13) and adipogenesis and osteogenesis are known to be inversely related (43, 44), we hypothesized a role of *ASCC1* promoting the regulation of MSC differentiation into osteoblasts at the expense of adipogenesis. In accordance, we observed in patient-derived fibroblasts a markedly reduced expression of two major regulators of early bone development and osteogenesis *SERPINF1* encoding pigment epithelium-derived factor (PEDF) (45) and *RUNX2*, the master transcriptional regulator in osteoblast differentiation (46). Our findings are in line with those of the first reported cases of children harboring a loss-of-function mutation in *ASCC1*, which showed reduced expression of genes involved in bone metabolism and neurogenesis including SERPINF1 in mutant fibroblasts (6). It should be underlined that PEDF is a multifunctional protein also involved in neuroprotection and neurogenesis. Thus, the previously observed downregulation of *SERPINF1* was primary linked to the neuromuscular disturbances and spinal muscular atrophy in affected patients rather than to bone fragility (6, 47). PEDF however is also a potent modulator of osteogenesis. It is temporally expressed by chondrocytes and osteoblasts during mouse bone development at sites of endochondral ossification and bone remodeling (48). Therefore, a decreased expression of PEDF might potentially impact bone remodeling and proper lamellar bone formation. Loss-of-function mutations in *SERPINF1* lead to osteogenesis imperfecta type VI and to defective matrix mineralization with increased osteoid formation and hypermineralization, a phenotype that however differs from the present case, although collagen fibril organization is also highly disordered in both of these patients (49, 50). Moreover, *SERPINF1* not only promotes osteoblast differentiation from MSCs but further plays a crucial role as a negative regulator of adipogenesis by suppressing adipogenic markers such as CEBPA, PPARγ, and ADIPOQ (45). Conversely, mice deficient in PEDF demonstrate increased adiposity (45). These studies suggest that mutated ASCC1 might have a stimulatory effect on adipogenic differentiation of MSCs due to downregulated *SERPINF1*. An important pathway in MSC differentiation during skeletal development, bone formation, and bone homeostasis is the TGF-β/SMAD pathway. The absence of SMAD3 leads to low bone mass disorders due to increased osteoblast apoptosis and the inability of osteoblasts to balance osteoclast activity in mice (51). Here, we demonstrated that stimulating the patient cells by exogenous TGF-β1 resulted in a diminished activation of SMAD3 with reduced levels of phosphorylated SMAD3 compared with the control cells. This emphasizes again a potential involvement of *ASCC1* in the regulation of osteogenesis.

The role of *ASCC1* in regulating osteogenic and adipogenic differentiation was further confirmed by our cell culture experiments using hMSCs in which *ASCC1* expression was knocked down by using two different shRNAs. *In vitro* differentiation of these cells into osteoblasts resulted in a marked reduction in calcium deposition, as demonstrated by Alizarin Red S staining, which coincided with the suppression of the prototypical osteogenic transcription factor *ALPL*. Conversely, the adipogenic drive of hMSCs was promoted, leading to a pronounced formation of lipid droplets, as visualized by Oil Red O staining, accompanied by upregulation of adipogenic markers including *PPARγ* and *FASN* (**Figure 6**).

The combined use of both shRNAs at the same time was not tested. However, based on our previous experience with this system, we expect that the combined use of two shRNAs will yield a similar effect as the best acting individual one.

This paper describes *ASCC1* as a crucial pro-osteogenic and anti-adipogenic factor in MSC differentiation. Our results support the results of the genome-wide association study by Cho et al. showing an association between *ASCC1* variants and an increased risk for osteoporosis and obesity in postmenopausal women (11). However, further studies are required to investigate the molecular mechanisms and potential interaction partners by which *ASCC1* modulates the MSC differentiation process. Indeed, it is still not fully clear how the present *ASCC1* deletion mutation is affecting the differentiation process. However, since this novel mutation leads to a more distinct downregulation of *ASCC1* than in the shRNA-mediated knockdown of *ASCC1* cells, we would expect an even stronger impact on osteogenesis and adipogenesis.

In conclusion, we have successfully identified a novel homozygous deletion mutation in *ASCC1* leading to a loss of function with multisystemic consequences. The recessive mutation affects the osteogenic and adipogenic differentiation capacity of hMSCs resulting in a distinct bone phenotype of disorganized, immature bone, and high bone marrow adiposity with impaired TGF-β/SMAD signaling. Thus, we can assign a new biological role to *ASCC1* acting as a molecular switch on the balance between osteogenesis and adipogenesis, which might also contribute to the understanding of common bone diseases such as osteoporosis as well as skeletal aging itself.

## Data availability statement

The original contributions presented in the study are included in the article/**Supplementary Material**. The ASCC1 variant is deposited in the ClinVar repository, accession number SUB13085728 (available at https://www.ncbi.nlm.nih.gov/clinvar/variation/2500145/). The complete exome sequencing data are not readily available because publication is not included in the consent for clinical exome sequencing. Requests to access the dataset in more detail should be directed to the corresponding author.

## Ethics statement

The studies involving human participants were reviewed and approved by Ethics commission of the Johannes Kepler University (reference number: 1149/2020) and the ethics committee Salzburg (reference number: 415-E/2552/10-2019). The post-mortem study providing control bone sample was approved by the East Midlands Research Ethics Committee (reference number: 17/EM/0061). The parents/guardian of the participants gave written informed consent to participate in this study. 

## Author contributions

BV provided the first draft of the manuscript and conducted statistical analysis. AE-G and WH designed the project and interpreted the data. JM, SW, BC, and WH provided the clinical data and arranged for the skin and bone tissue samples. SU collected the bone sample from the age-matched control infant. NF-Z and SB performed and interpreted histomorphometry and qBEI analyses. BV performed PCR and qPCR and, together with HH, immunoblotting experiments. RK and HP performed and analyzed the WES, RNA sequencing, and proteomics data. MK and BE performed the differentiation assays including Alizarin S stain, Oil Red O stain, calcium quantification, and qPCR. All authors provided intellectual input and critically reviewed the manuscript. All authors contributed to the article and approved the submitted version.
