# Supplementary material for: Investigating the role of ASCC1 in the causation of bone fragility
Source: Front Endocrinol (Lausanne). 2023 Jun 30;14:1137573. doi: 10.3389/fendo.2023.1137573 (PMC10348481; doi:10.3389/fendo.2023.1137573)
Supplement: Supplementary file 1 [file DataSheet_1.pdf]

Supplementary Material

1 Supplementary Tables

Supplementary Table 1. Forward and reverse primer sequences of the analyzed genes and sequences of the shRNAs used for *ASCC1*

**Gene**	**Forward Primer (5'-3')**	**Reverse Primer (5'-3')**
36B4	GTCCTCGTGGAAGGCCC	AGGAGAGACAGGGAGCTCAG
ASCC1	GCTTCCCTCACGGGCATAAT	TAAGCTGTGGACGCAGAACT
ALPL	CTATCCTGGCTCCGTGCTC	ACTGATGTTCCAATCCTGCG
RUNX2	GATTACAGACCCCAGGCAGG	GGCTCAGGTAGGAGGGGTAA
CTNNB1	CGGTCGGACTCCCGC	TCCAACTCCATCAAATCAGCTTG
FASN	CAACCTCTCCCAGGTATGCG	CCAGGGAGCTGTGGATGATG
PPARG	TCTCAAACGAGAGTCAGCCT	CACGGAGCTGATCCCAAAGT
CEBPA	TGATGCCGTTCACACACTTCA	CAAGGCCATAAGGCACTGC
**shRNA name**	**Target & Flanking Sequence**	
ID12209 [#1 shRNA ASCC1]	CCGG**CAAGAACAGACCTATCAACAT**CTCGAGATGTTGATAGGTCTGTTCTTGTTTTTTG	
ID12211 [#2 shRNA ASCC1]	CCGG**GAAGATGAAGAGGACTTCTAT**CTCGAGATAGAAGTCCTCTTCATCTTCTTTTTTG	

2 Supplementary Figures

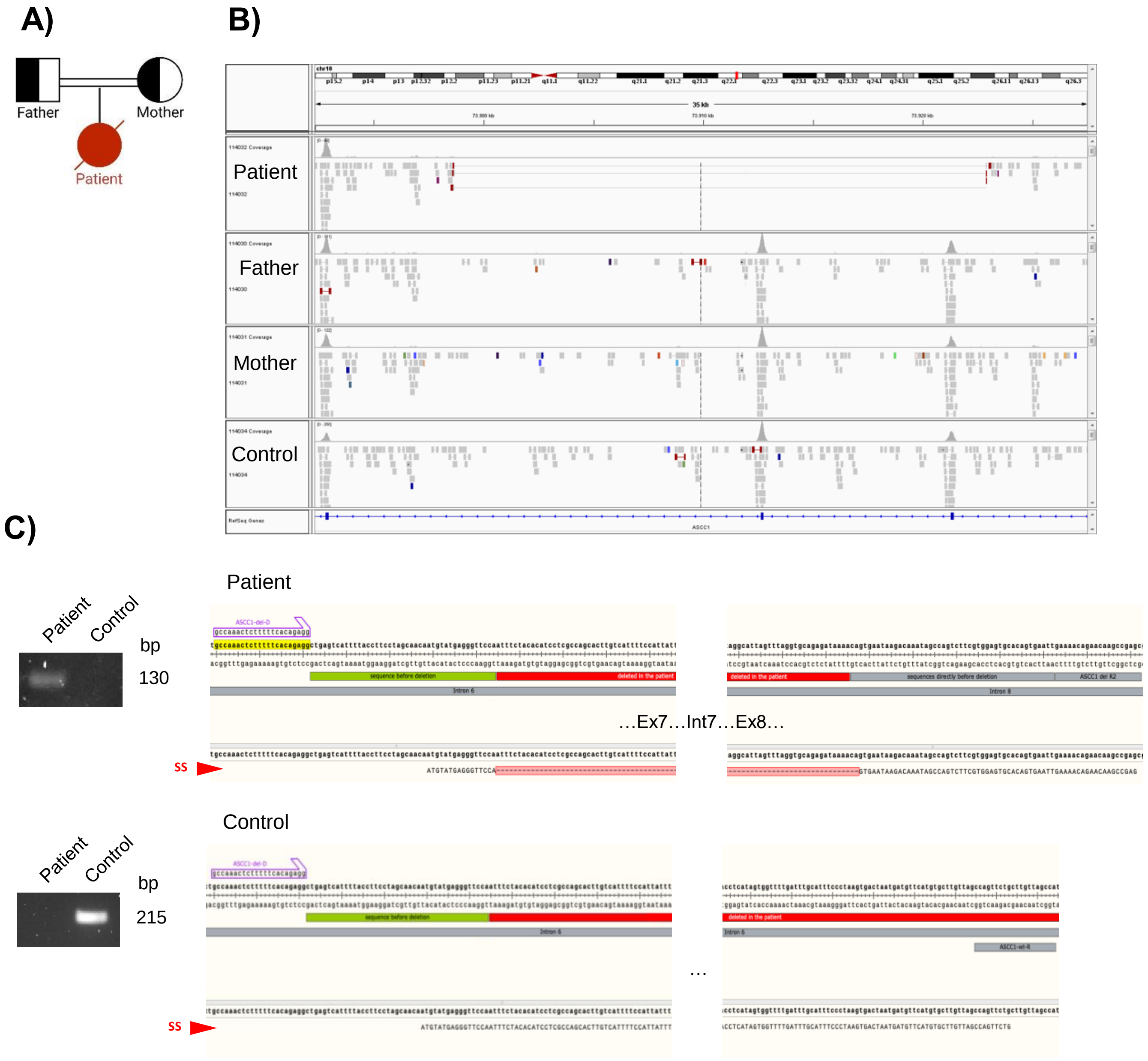

Supplementary Figure 1. Homozygous deletion in *ASCC1*. A) Family pedigree with *ASCC1* homozygosity in the patient and heterozygosity in the parents. Two horizontal lines between father and mother represent consanguineous mating. B) Exome sequencing displaying the *ASCC1* variant (NM_001198799.2: c.[574-1471_831-5742del24223];[574-1471_831-5742del24223]) in the homozygous state in the patient C) Verification of the ASCC1 mutation in the patient-derived skin fibroblasts and of an intact ASCC1 sequence in the healthy control cells by Sanger sequencing. “SS” shows the output of Sanger sequencing.

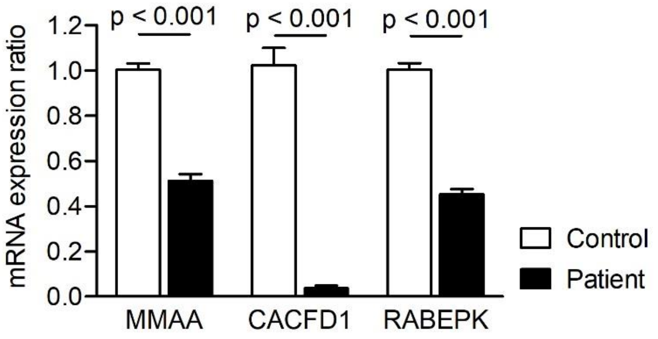

Supplementary Figure 2. Quantitative PCR showing mRNA expression ratio of MMAA, CACFD1 and RABEPK in age-, gender- and passage-matched patient and control fibroblasts. Target genes were normalized to HPRT1. Columns represent the mean of at least three independent experiments and error bars show standard error of the mean (SEM). The p values were calculated using a two-tailed t test.

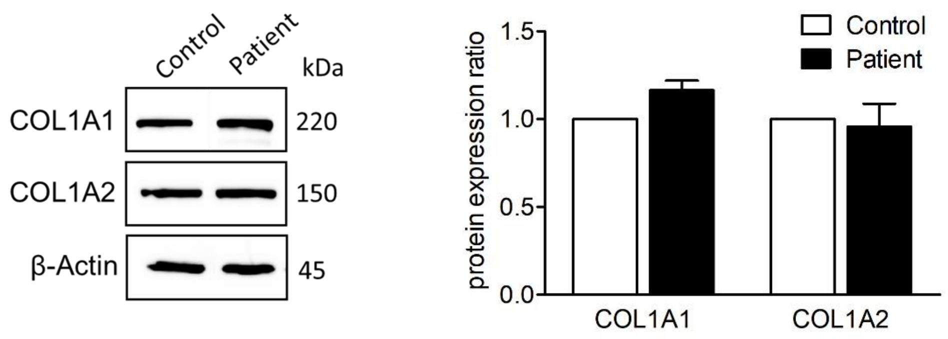

Supplementary Figure 3. Protein expression analysis of COL1A1 and COL1A2 in patient and control cells by western blotting. All target proteins were normalized to β-Actin. Columns represent the mean of three independent experiments and error bars show standard error of the mean (SEM).

Information on downregulated/upregulated genes not involved in bone metabolism:

These downregulated genes include *ASCC2*, a subunit of the ASC-1 complex (1), CACFD1, which functions as a calcium channel to regulate synaptic endocytosis (2), RABEPK which is required for endosome to trans-Golgi network transport (3), MMAA which is involved in Vitamin B12 metabolism and transport into the mitochondria (4, 5), ESD which is a serine hydrolase involved in the detoxification of formaldehyde (6, 7), and BDH2, a short chain dehydrogenase/reductase family member which catalyzes a rate-limiting step in the regulation of intracellular iron metabolism and siderophore biogenesis (8, 9). In addition, NDNF, a neurotrophic factor that plays a role in various cellular processes including neuron migration, growth and survival and olfactory development (10, 11) was upregulated.

3 REFERENCES

1. Jung D-J, Sung H-S, Goo Y-W, Lee HM, Park OK, Jung S-Y, et al. Novel transcription coactivator complex containing activating signal cointegrator 1. *Molecular and cellular biology* (2002) **22**:5203–11. doi:10.1128/MCB.22.14.5203-5211.2002.
2. Yao C-K, Lin YQ, Ly CV, Ohyama T, Haueter CM, Moiseenkova-Bell VY, et al. A synaptic vesicle-associated Ca2+ channel promotes endocytosis and couples exocytosis to endocytosis. *Cell* (2009) **138**:947–60. doi:10.1016/j.cell.2009.06.033.
3. Díaz E, Schimmöller F, Pfeffer SR. A novel Rab9 effector required for endosome-to-TGN transport. *The Journal of cell biology* (1997) **138**:283–90. doi:10.1083/jcb.138.2.283.
4. Froese DS, Kochan G, Muniz JR, Wu X, Gileadi C, Ugochukwu E, et al. Structures of the human GTPase MMAA and vitamin B12-dependent methylmalonyl-CoA mutase and insight into their complex formation. *The Journal of biological chemistry* (2010) **285**:38204–13. doi:10.1074/jbc.M110.177717.
5. Plessl T, Bürer C, Lutz S, Yue WW, Baumgartner MR, Froese DS. Protein destabilization and loss of protein-protein interaction are fundamental mechanisms in cblA-type methylmalonic aciduria. *Human mutation* (2017) **38**:988–1001. doi:10.1002/humu.23251.
6. Eiberg H, Mohr J. Identity of the polymorphisms for esterase D and S-formylglutathione hydrolase in red blood cells. *Human genetics* (1986) **74**:174–5. doi:10.1007/BF00282085.
7. Hopkinson DA, Mestriner MA, Cortner J, Harris H. Esterase D: a new human polymorphism. *Annals of human genetics* (1973) **37**:119–37. doi:10.1111/j.1469-1809.1973.tb01820.x.
8. Guo K, Lukacik P, Papagrigoriou E, Meier M, Lee WH, Adamski J, et al. Characterization of human DHRS6, an orphan short chain dehydrogenase/reductase enzyme: a novel, cytosolic type 2 R-beta-hydroxybutyrate dehydrogenase. *The Journal of biological chemistry* (2006) **281**:10291–7. doi:10.1074/jbc.M511346200.
9. Devireddy LR, Hart DO, Goetz DH, Green MR. A mammalian siderophore synthesized by an enzyme with a bacterial homolog involved in enterobactin production. *Cell* (2010) **141**:1006–17. doi:10.1016/j.cell.2010.04.040.
10. Kuang X-L, Zhao X-M, Xu H-F, Shi Y-Y, Deng J-B, Sun G-T. Spatio-temporal expression of a novel neuron-derived neurotrophic factor (NDNF) in mouse brains during development. *BMC neuroscience* (2010) **11**:137. doi:10.1186/1471-2202-11-137.
11. Messina A, Pulli K, Santini S, Acierno J, Känsäkoski J, Cassatella D, et al. Neuron-Derived Neurotrophic Factor Is Mutated in Congenital Hypogonadotropic Hypogonadism. *American journal of human genetics* (2020) **106**:58–70. doi:10.1016/j.ajhg.2019.12.003.
